# ACO-CLS: Ant Colony Optimization-Based Collaborative Localization and Search for Multi-Robot Systems

**DOI:** 10.3390/s26092831

**Published:** 2026-05-01

**Authors:** Zhengyang He, Xiaojie Tang, Fengyun Zhang

**Affiliations:** 1 School of Intelligent Manufacturing, Sichuan University Jinjiang College, Meishan 620860, China; hezhengyang@scujj.edu.cn (Z.H.); tangxiaojie@scujj.edu.cn (X.T.); 2 College of Artificial Intelligence, Southwest University, Chongqing 400715, China

**Keywords:** multi-robot system, collaborative localization, multi-sensor fusion, ant colony optimization, intelligent search, dynamic grouping

## Abstract

With the rapid development of robot technology, the multi-robot cooperation system has been widely used in rescue, monitoring, logistics, and other fields. Aiming at the key problems in multi-robot cooperative localization and target search, considering the search time, search mileage, and search risk, a cooperative localization and search algorithm based on ant colony optimization (ACO-CLS) is proposed based on the analysis of the target weight factor, the sensitivity of the number of robots, the adaptability of robot formation, and the sensitivity of robot speed. Firstly, a multi-sensor fusion localization algorithm based on IMU and UWB sensors is designed, and the error-state Kalman filter (ESKF) is used to achieve high-precision position estimation. Secondly, a dynamic grouping strategy based on weight is proposed to realize intelligent grouping based on target priority and robot position. Then, the ant colony algorithm is introduced to make path decisions, and the robot search is guided by pheromone updates and heuristic information. Finally, an intelligent reallocation mechanism after target discovery is designed to realize the dynamic optimization of resource allocation. The simulation results show that the proposed algorithm is superior to the traditional methods in terms of location accuracy, search efficiency, and system robustness, and has important theoretical value and application prospects.

## 1. Introduction

Multi-robot systems (MRSs) have attracted increasing attention due to their superior adaptability, efficiency, and robustness compared to single-robot platforms. They are widely employed in complex and dynamic environments for tasks such as search and rescue, environmental monitoring, and logistics [1,2]. In such applications, two fundamental challenges must be addressed simultaneously: accurate self-localization in GPS-denied environments and efficient collaborative target search. Localization provides the necessary spatial awareness for coordination, while search algorithms enable robots to locate objects of interest in unknown areas [3,4]. However, achieving both high localization accuracy and rapid search remains difficult due to sensor limitations, communication constraints, and environmental uncertainties.

For robot localization in GPS-denied environments, Lin et al. [5] proposed an improved error-state Kalman filter (ESKF) algorithm that fuses UWB, IMU, and barometer data to achieve accurate indoor positioning. Sun et al. [6] developed a UWB/IMU/odometer-based localization system that maintains high accuracy under both LOS and NLOS mixed conditions. For multi-robot task allocation, Zhang et al. [7] introduced an opinion-based distributed strategy enabling scalable coordination in robot swarms without centralized control. Martin et al. [8] proposed an iterative clustering approach specifically designed for heterogeneous multi-robot systems to achieve effective collaborative task distribution. For path planning in multi-robot systems, Huang et al. [9] combined an improved ant colony optimization (ACO) algorithm with the dynamic window approach, achieving both global optimality and local obstacle avoidance for delivery robots. Yu et al. [10] enhanced ACO performance through adaptive parameter tuning, demonstrating improved convergence for mobile robot path planning. Dong et al. [11] developed an adaptive hybrid response mechanism for dynamic multi-objective optimization in multi-robot task allocation problems. Paul and Chowdhury [12] leveraged capsule networks combined with attention mechanisms to learn complex task allocation policies in unknown environments. Zhonghao Lyu proposes quantization-aware collaborative inference with joint bit-width and frequency optimization to balance inference distortion, latency, and energy for large AI models on resource-constrained embodied agents [13]. Mendonca, Marcio compares three swarm-based rescue robot controllers—FLC, DFCM, and DFCM-ACO—finding that DFCM-based methods reduce processing time and travel distance, with DFCM-ACO offering the best overall balance [14].

While these advances have significantly improved individual aspects of MRS, an integrated framework that simultaneously addresses high-precision localization, dynamic task allocation, and intelligent path planning remains limited. This paper proposes a comprehensive multi-robot collaborative system that combines ESKF-based UWB/IMU localization, weight-based dynamic grouping for task allocation, ACO-based path planning enhanced with pheromone mechanisms, and an intelligent reallocation strategy for post-discovery resource optimization. Simulation results demonstrate that the proposed integrated approach achieves superior performance in localization accuracy, search efficiency, and system robustness.

This paper proposes an integrated algorithmic framework for multi-robot collaborative localization and target search. The core of the approach consists of four key components: first, a high-precision localization algorithm that fuses data from IMU and UWB sensors using an error-state Kalman filter (ESKF). Second, a weight-based dynamic grouping strategy enables intelligent robot team formation according to target priority and real-time robot positions. Third, path planning and search guidance are optimized by employing the ant colony optimization (ACO) algorithm, which leverages pheromone updating and heuristic information to efficiently direct robot movement. Finally, an intelligent reallocation mechanism dynamically redistributes resources once a target is found. Together, these algorithms enhance overall system performance in localization accuracy, search efficiency, and robustness compared to conventional methods.

The main contributions of this paper include:

(1) **ESKF:** Fuses IMU and UWB measurements for error-state filtering, suppresses sensor noise and bias drift, achieves high-precision pose estimation, and provides reliable position feedback for ant colony search.
(2) **K-means clustering:** Performs dynamic grouping based on initial robot positions and target weights, re-clusters after a target is found, adaptively adjusts the size of each group to balance search resources, and improves collaborative efficiency.
(3) **ACO:** Each group maintains a pheromone map; robots move based on pheromone concentration and inverse distance to target, with an exploration factor balancing exploration and exploitation. Target discovery triggers strong pheromone release, guiding group convergence for efficient multi-target search.

The structure of this paper is as follows: Section 2 introduces related work; Section 3 describes the system framework design; Section 4 details the robot localization algorithm; Section 5 presents the intelligent search and search algorithm; Section 6 introduces the experimental model; Section 7 shows simulation and results analysis; Section 8 concludes the paper and discusses future work.

## 2. Related Work

Accurate self-localization is a prerequisite for any cooperative behavior in multi-robot systems. In GPS-denied environments, robots must rely on onboard sensors and inter-robot measurements. In the field of multi-sensor fusion localization, Sun et al. [15] proposed a simultaneous calibration and localization framework integrating UWB, IMU, and odometer data, while Tran and Ryoo [16] developed a comprehensive multi-sensor fusion system combining UWB, odometry, and AHRS for reliable mobile robot trajectory tracking. For collaborative Simultaneous Localization and Mapping (SLAM), Lajoie and Beltrame [17] introduced a sparse decentralized framework enabling efficient collaborative mapping in multi-robot systems, and Cao et al. [18] proposed a distributed variational inference approach for multi-robot object SLAM. In relative localization, Liang et al. [19] developed a 3D relative localization method using angle and self-displacement measurements, and Chen et al. [20] established a theoretical framework for relative localizability in multi-robot systems.

For multi-robot task allocation, Zhang et al. [21] presented a dynamic and prioritized scheduling method using deep reinforcement learning for heterogeneous systems, while Verma et al. [22] proposed a coalition formation framework tailored to heterogeneous multi-robot task allocation.

In exploration and path planning, Ning et al. [23] introduced a hybrid multi-strategy rapidly exploring random tree algorithm for collaborative exploration in unknown environments, and Chakraborty and Sahu [24] developed an improved path planning algorithm with optimization capabilities for mobile robots. Swarm intelligence algorithms have also been widely adopted. Liu et al. [25] proposed an adaptive dual-layer ant colony optimization algorithm integrated with a dynamic window approach for robot path planning, and Li et al. [26] developed a multi-strategy genetic ant colony optimization algorithm for comprehensive robot path planning. For grey wolf optimizer applications, Zhang et al. [27] introduced a hybrid tabu–grey wolf optimizer algorithm for cold-chain logistics distribution optimization, and Gai et al. [28] developed a leader–follower hybrid particle swarm–grey wolf optimizer for constrained UAV path planning.

In summary, existing research has made significant strides in multi-robot localization, task allocation, and path planning. However, most approaches treat these problems separately, leading to suboptimal overall performance. Localization accuracy directly impacts the effectiveness of task allocation and path planning, yet few frameworks integrate high-precision localization with search algorithms in a closed loop. This paper bridges this gap by proposing an integrated framework that combines ESKF-based UWB/IMU localization, dynamic grouping, ACO-based path planning, and intelligent reallocation. The synergy among these components enables robust and efficient target search in GPS-denied environments, as demonstrated through extensive simulations.

## 3. System Framework Design

**Figure 1:**
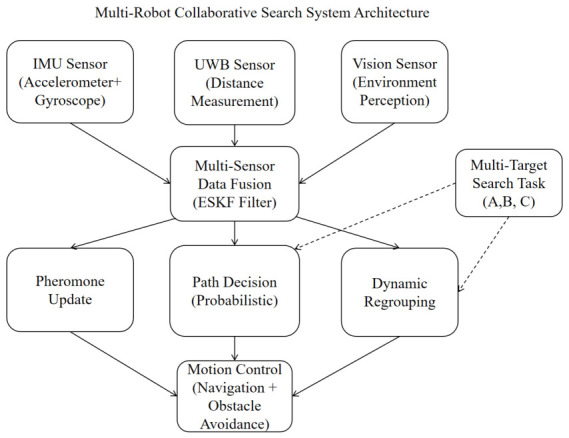
Multi-robot collaborative search system architecture.

This section introduces the overall framework design of the multi-robot collaborative search system, including system architecture, functional modules, and workflow, as shown in Figure 1. The positioning information is obtained through the ESKF information fusion of the IMU and the UWB. The visual sensor in the system diagram is actually an ordinary camera. Usually, the pre-trained ResNet50 can be used to recognize the image of the camera to determine whether it is a target. Then the robot formation information is updated in real time, the path planning is carried out according to different bionic algorithms, the dynamic reorganization is completed according to whether the target is found, and finally, the motion control of the robot is implemented.

### 3.1. Multi-Robot Collaborative Search Framework Based on Ant Colony Algorithm

The multi-robot collaborative search system proposed in this paper mainly consists of the following core modules:

(1) **Sensor Layer:** Includes IMU and UWB sensors. The IMU provides acceleration and angular velocity information, while UWB provides distance information to anchors. These two sensors complement each other: IMU provides high-frequency relative position changes, and UWB provides absolute position reference.
(2) **Data Fusion Layer:** Uses an error-state Kalman filter (ESKF) to fuse IMU and UWB data, achieving high-precision position estimation. ESKF performs linearization in the state space, avoiding nonlinear issues, making it suitable for real-time applications.
(3) **Ant Colony Algorithm Layer:** The core decision-making module, including functions such as pheromone updates, path decision-making, and dynamic regrouping. Through the positive feedback mechanism of pheromones, it guides robots to converge towards target areas.
(4) **Control Layer:** Controls robot motion based on the decisions from the ant colony algorithm, including navigation and obstacle avoidance.

### 3.2. Dynamic Grouping and Adaptive Search Strategy

The system employs a dynamic grouping strategy, dividing multiple robots into different search groups based on target priority and position distribution. Each group is responsible for searching one or more targets, and within-group collaboration is achieved through information sharing. Advantages of the grouping strategy include: (1) improving search efficiency by avoiding robots repeatedly searching the same area; (2) enhancing system robustness, as failure of a single robot does not affect overall task execution; (3)supporting parallel search, reducing task completion time.

### 3.3. Precise Localization via Hybrid Sensor Fusion

To achieve high-precision robot localization, this paper adopts a hybrid fusion scheme combining IMU and UWB. IMU has high frequency and low latency but suffers from integration drift; UWB has absolute positioning capability but lower update frequency. By fusing the advantages of both sensors using ESKF, high-precision and highly reliable position estimation is achieved.

### 3.4. Intelligent Reallocation Mechanism After Target Discovery

When a target is discovered, the system needs to reallocate resources. This paper designs an intelligent reallocation mechanism: (1) select one robot as a companion robot to remain at the target location; (2) reassign other robots from that group to search for remaining targets; (3) update the pheromone map, releasing a large amount of pheromones to guide other robots to the location.

## 4. Robot Localization Algorithm

This chapter details the robot localization algorithm based on multi-sensor fusion, including IMU localization, UWB localization, and ESKF data fusion.

### 4.1. IMU Localization Algorithm

An Inertial Measurement Unit (IMU) includes an accelerometer and a gyroscope, measuring the robot’s linear acceleration and angular velocity. Position information can be obtained by double-integrating acceleration, and attitude information can be obtained by single-integrating angular velocity.

The following is an integral positioning model in continuous time neglecting the effect of measurement noise:

(1) $$\left\{\begin{array}{c} \dot{R}=R{\left(\tilde{\omega }-b_{g}\right)}^{\wedge },\\ \dot{p}=v,\\ \dot{v}=R\left(\tilde{a}-b_{a}\right)+g, \end{array}\right.\;\mathrm{where}\;\left\{\begin{array}{c} \tilde{\omega }=\omega +b_{g},\\ \tilde{a}=R^{T}\left(a-g\right)+b_{a}. \end{array}\right.$$

where *R* represents the rotation part of the robot during movement and is a rotation matrix; $\tilde{\omega }$ represents the instantaneous angular velocity of the robot at a certain moment; $b_{g}$ and $b_{a}$ represent the zero bias of the gyroscope and the accelerometer, respectively; and *g* represents gravitational acceleration. $\tilde{a}$ represents the acceleration of the robot in the vehicle coordinate system, and *p* and *v* represent the position and velocity of the robot in the world coordinate system, respectively.

### 4.2. UWB Localization Algorithm

Ultra-Wideband (UWB) is a wireless communication technology with high time resolution, enabling high-precision distance measurement. By measuring the distances from the robot to multiple anchors, the robot’s position can be estimated via trilateration or least-squares methods.

By using distance measurements from at least four anchors, the robot’s 3D position can be calculated. Advantages of UWB localization are absolute positioning and no integration drift; disadvantages are lower update frequency and susceptibility to NLOS propagation.

The following equation set is listed in order to realize the positioning of point *P*:

(2) $$\left\{\begin{array}{c} {\left(x-x_{1}\right)}^{2}+{\left(y-y_{1}\right)}^{2}+{\left(z-z_{1}\right)}^{2}=d_{1}^{2},\\ {\left(x-x_{2}\right)}^{2}+{\left(y-y_{2}\right)}^{2}+{\left(z-z_{2}\right)}^{2}=d_{2}^{2},\\ \;\vdots \\ {\left(x-x_{n}\right)}^{2}+{\left(y-y_{n}\right)}^{2}+{\left(z-z_{n}\right)}^{2}=d_{n}^{2}. \end{array}\right.$$

An equation of the form AX=b is obtained by Gaussian elimination:

(3) $$2A\left[\begin{array}{c} x\\ y\\ z \end{array}\right]=-\left[\begin{array}{c} d_{1}^{2}-d_{n}^{2}\\ d_{2}^{2}-d_{n}^{2}\\ \vdots \\ d_{n-1}^{2}-d_{n}^{2} \end{array}\right]+c,$$

where

(4) $$A=\left[\begin{array}{ccc} x_{1}-x_{n} & y_{1}-y_{n} & z_{1}-z_{n}\\ x_{2}-x_{n} & y_{2}-y_{n} & z_{2}-z_{n}\\ \vdots & \vdots & \vdots \\ x_{n-1}-x_{n} & y_{n-1}-y_{n} & z_{n-1}-z_{n} \end{array}\right],$$

and therefore, the least-squares estimate is

(5) $$\left[\begin{array}{c} x\\ y\\ z \end{array}\right]=-\frac{1}{2}{\left(A^{T}A\right)}^{-1}A^{T}\left(\left[\begin{array}{c} d_{1}^{2}-d_{n}^{2}\\ d_{2}^{2}-d_{n}^{2}\\ \vdots \\ d_{n-1}^{2}-d_{n}^{2} \end{array}\right]-c\right).$$

Finally, the position coordinates of the target point can be obtained by using the minimum mean square error estimation. In this paper, the robot cluster search is carried out in a ground environment, so the coordinate *z* of the robot is taken as 0, where *A* is the difference between the coordinates of each anchor point, and *c* is a constant associated with each anchor coordinate.

### 4.3. ESKF Filter Design Based on IMU and UWB

To combine the advantages of IMU and UWB, this paper uses an error-state Kalman filter (ESKF) for data fusion. ESKF performs linearization in the error-state space, avoiding the nonlinear problems of traditional EKF.

In the project, the IMU is treated as a motion model and the UWB-EKF is treated as an observation model. The following is the inference procedure for achieving localization using error-state Kalman filtering.

First, define the nominal state as

(6) $$\hat{x}={\left[\hat{p},\;\hat{v},\;\hat{R},\;{\hat{b}}_{g},\;{\hat{b}}_{a},\;\hat{g}\right]}^{T},$$

where $\hat{p}$ is translation, $\hat{v}$ is velocity, and $\hat{R}$ is rotation. ${\hat{b}}_{g}$ and ${\hat{b}}_{a}$ are the gyroscope and accelerometer bias estimates, respectively, and $\hat{g}$ is gravity. The nominal-state dynamics in continuous time are written as follows:

(7) $$\left\{\begin{array}{c} {\dot{\hat{p}}}_{t}={\hat{v}}_{t},\\ {\dot{\hat{v}}}_{t}={\hat{R}}_{t}\left(a_{t}-{\hat{b}}_{a,t}-\eta_{a}\right)+{\hat{g}}_{t},\\ {\dot{\hat{R}}}_{t}={\hat{R}}_{t}{\left(\omega_{t}-{\hat{b}}_{g,t}-\eta_{g}\right)}^{\wedge },\\ {\dot{\hat{b}}}_{g,t}=\eta_{b_{g}},\\ {\dot{\hat{b}}}_{a,t}=\eta_{b_{a}},\\ {\dot{\hat{g}}}_{t}=0. \end{array}\right.$$

The error state is then defined by the perturbation relationship

(8) $$\delta x={\left[\delta p,\;\delta v,\;\delta \theta ,\;\delta b_{g},\;\delta b_{a},\;\delta g\right]}^{T},$$

with

(9) $$\left\{\begin{array}{c} p_{t}={\hat{p}}_{t}+\delta p_{t},\\ v_{t}={\hat{v}}_{t}+\delta v_{t},\\ R_{t}=\exp\left({\left(\delta \theta_{t}\right)}^{\wedge }\right){\hat{R}}_{t},\\ b_{g,t}={\hat{b}}_{g,t}+\delta b_{g,t},\\ b_{a,t}={\hat{b}}_{a,t}+\delta b_{a,t},\\ g_{t}={\hat{g}}_{t}+\delta g_{t}. \end{array}\right.$$

By taking the time derivative on both sides of the above equation and completing the inference procedure of the rotation and velocity terms of the error state, the following equation of motion for the error variable can be obtained:

(10) $$\left\{\begin{array}{c} \delta \dot{p}=\delta v,\\ \delta \dot{v}=-\hat{R}{\left(a-{\hat{b}}_{a}\right)}^{\wedge }\delta \theta -\hat{R}\;\delta b_{a}-\eta_{a}+\delta g,\\ \delta \dot{\theta }=-{\left(\omega -{\hat{b}}_{g}\right)}^{\wedge }\delta \theta -\delta b_{g}-\eta_{g},\\ \delta {\dot{b}}_{g}=\eta_{b_{g}},\\ \delta {\dot{b}}_{a}=\eta_{b_{a}},\\ \delta \dot{g}=0. \end{array}\right.$$

If the above equation is written in differential form, the following expression is obtained:

(11) $$\left\{\begin{array}{c} \delta p(t+\Delta t)=\delta p(t)+\delta v(t)\;\Delta t,\\ \delta v\left(t+\Delta t\right)=\delta v\left(t\right)+\left(-\hat{R}{\left(a-{\hat{b}}_{a}\right)}^{\wedge }\delta \theta -\hat{R}\;\delta b_{a}+\delta g\right)\Delta t-\eta_{a}\;\Delta t,\\ \delta \theta \left(t+\Delta t\right)=\mathrm{Exp}\left(-(\omega -{\hat{b}}_{g})\Delta t\right)\;\delta \theta \left(t\right)-\delta b_{g}\;\Delta t-\eta_{g}\;\Delta t,\\ \delta b_{g}\left(t+\Delta t\right)=\delta b_{g}\left(t\right)+\eta_{b_{g}}\;\Delta t,\\ \delta b_{a}\left(t+\Delta t\right)=\delta b_{a}\left(t\right)+\eta_{b_{a}}\;\Delta t,\\ \delta g(t+\Delta t)=\delta g(t). \end{array}\right.$$

Note that the right-hand side of the above equation omits (t) in parentheses to simplify the equation.

The motion process of the ESKF is then written in discrete time, where δx is the error-state variable:

(12) $$\delta x_{k+1}=f\left(\delta x_{k}\right)+w_{k},\;w_{k}∼N\left(0,Q\right).$$

Here, $w_{k}$ denotes process noise, and *Q* can be written in diagonal form as

(13) $$Q=\mathrm{diag}\left(0_{3},\;\mathrm{Cov}\left(\eta_{v}\right),\;\mathrm{Cov}\left(\eta_{\theta }\right),\;\mathrm{Cov}\left(\eta_{g}\right),\;\mathrm{Cov}\left(\eta_{a}\right),\;0_{3}\right).$$

where $\eta_{v}$ and $\eta_{\theta }$ are the measurement noises of the robot velocity and rotation angle, respectively. $\eta_{g}$ and $\eta_{a}$ are the measurement noises of the gyroscope and accelerometer, respectively.

In the calculation, the linearized form of the equation of motion is calculated as follows:

(14) $$\delta x\left(t+\Delta t\right)\approx f\left(\delta x(t)\right)+F_{t}\;\delta x\left(t\right)+w_{t}.$$

where $F_{t}$ is the linearized Jacobian matrix. A standard first-order discretization is

(15) $$F_{t}=\left[\begin{array}{cccccc} I & I\Delta t & 0 & 0 & 0 & 0\\ 0 & I & -\hat{R}{\left(a-{\hat{b}}_{a}\right)}^{\wedge }\Delta t & 0 & -\hat{R}\Delta t & I\Delta t\\ 0 & 0 & \mathrm{Exp}\left(-(\omega -{\hat{b}}_{g})\Delta t\right) & -I\Delta t & 0 & 0\\ 0 & 0 & 0 & I & 0 & 0\\ 0 & 0 & 0 & 0 & I & 0\\ 0 & 0 & 0 & 0 & 0 & I \end{array}\right].$$

Based on the above basis, the prediction of the ESKF is performed, which contains the prediction of the nominal state (IMU integral) and the prediction of the error state:

(16) $$\delta x_{\mathrm{pred}}=F_{t}\;\delta x,\;P_{\mathrm{pred}}=F_{t}PF_{t}^{T}+Q.$$

Since the error state of the ESKF is reset to 0 after each update, the mean part of the equation of motion is not very meaningful. But the covariance part describes the distribution of the entire error estimate. Next is the update process of the ESKF. Assuming that the UWB sensor is used for observation and its observation equation is h(x), the observation equation can be written as follows:

(17) z=h(x)+v,v∼N(0,V).

where *z* is the observed data, *v* is the observation noise, and *V* is the covariance matrix of this noise. In the traditional EKF, the observation equation is linearized directly with respect to the full state. In the ESKF, however, there is a nominal-state estimate $\hat{x}$ together with an error-state estimate δx. Therefore, the required Jacobian is the derivative of the observation model with respect to the error state:

(18) $$H={\left.\frac{\partial h}{\partial \delta x}\right|}_{{\hat{x}}_{\mathrm{pred}}}.$$

The Kalman gain is then computed, and the update process for the error state can be computed as follows:

(19) $$\left\{\begin{array}{c} K=P_{\mathrm{pred}}H^{T}{\left(HP_{\mathrm{pred}}H^{T}+V\right)}^{-1},\\ \delta x=K\left(z-h({\hat{x}}_{\mathrm{pred}})\right),\\ \hat{x}={\hat{x}}_{\mathrm{pred}}⊕\delta x,\\ P=\left(I-KH\right)P_{\mathrm{pred}}. \end{array}\right.$$

where *K* is the Kalman gain, $P_{\mathrm{pred}}$ is the predicted covariance matrix, and the last *P* is the corrected covariance matrix. After the prediction and update process is completed, the estimation of the error state is corrected. The error state needs to be integrated into the nominal state, and then the ESKF is reset.

### 4.4. Localization Error Compensation Mechanism

To further improve localization accuracy, this paper designs an error compensation mechanism. It mainly includes multi-robot collaborative calibration, performing cross-validation using relative position information between robots. Multi-robot cooperative calibration is essentially a process of distributed state estimation and error graph optimization. By transforming the relative position observations between robots into error constraints $e_{j}-e_{i}$, an interconnected error propagation network is constructed and the optimization problem is solved. The positioning error is transferred from the high-confidence robot to the low-confidence robot, and the average allocation of the error is realized when a closed loop is formed. Finally, the positioning accuracy of the whole robot team tends to be consistent and higher than that of a single robot.

#### 4.4.1. Problem Modeling

Suppose there are *n* robots moving in the same environment. For robot *i*, the true pose at time *t* is $X_{i}\left(t\right)$. Due to system error, the pose estimated by the robot through its own sensors is ${\hat{X}}_{i}\left(t\right)$. Define the deviation (error) of the estimated value from the true value as

(20) $$e_{i}\left(t\right)={\hat{X}}_{i}\left(t\right)-X_{i}\left(t\right).$$

The goal of collaborative calibration is to estimate and eliminate this bias $e_{i}\left(t\right)$ through relative measurements between robots.

#### 4.4.2. Relative Observation Equation

When robot *j* is observed by robot *i*, a relative position vector $Z_{ij}$ (ranging or direction finding) between them can be obtained.

1. The real situation:
  (21) $$Z_{ij}^{\mathrm{true}}=X_{j}-X_{i}.$$
2. Measurement case: robot *i* predicts the position of robot *j* based on its own positioning estimation and the relative information measured by the sensor:
  (22) $${\hat{X}}_{j}^{\left(i\right)}={\hat{X}}_{i}+Z_{ij}^{\mathrm{measured}},$$
  where $Z_{ij}^{\mathrm{measured}}$ is the actual measured relative vector.

#### 4.4.3. Consistency Constraints and Error Propagation

The position ${\hat{X}}_{j}^{\left(i\right)}$ of robot *j* predicted by robot *i* does not generally coincide with the position ${\hat{X}}_{j}$ estimated by robot *j* itself, and the consistency residual is defined as

(23) $$r_{ij}={\hat{X}}_{j}-{\hat{X}}_{j}^{\left(i\right)}.$$

Substituting the relationship between the estimated value and the true value, $X=\hat{X}-e$, and assuming that the relative measurement itself is more accurate, $Z_{ij}^{\mathrm{measured}}\approx X_{j}-X_{i}$, we get

(24) $$r_{ij}\approx e_{j}-e_{i}.$$

This shows that the residuals of the estimated positions of the two robots directly reflect the vector difference in their respective positioning errors. This is the central relation for cross-checking.

#### 4.4.4. Error Map Optimization

The overall system is modeled as a graph optimization problem:

1. Node: the pose ${\hat{X}}_{i}^{k}$ of each robot at different times.
2. IMU position prediction: connects the nodes of the same robot at adjacent times; constrains the relative motion, but with cumulative drift.
3. UWB cooperative observation edge: connects nodes of different robots at the same time and constrains their relative positions.
4. For a co-observation edge, the error function is defined as

(25) $$e_{ij}\left(X_{i},X_{j}\right)=\Theta \left(X_{j},X_{i}\right)-Z_{ij},$$

where Θ represents the operation of obtaining the relative pose. The goal of the overall system is to minimize the sum of squares of all error terms:

(26) $$\min_{X}\sum_{i}∥e_{i}^{\mathrm{odom}}{∥}^{2}+\sum_{i,j}{∥e_{ij}∥}^{2}.$$

Solving the optimization problem (Gauss–Newton method is used in this paper) will force the error term $r_{ij}\approx e_{j}-e_{i}$ between robots to be reduced, thus realizing cross-checking.

The IMU sensor in this program includes a three-axis accelerometer and a three-axis gyroscope, with noise characteristics of an accelerometer standard deviation of $0.05\;m/s^{2}$, a gyroscope standard deviation of 0.005 rad/s, and constant biases of $0.005\;m/s^{2}$ and 0.0005 rad/s, respectively. The UWB sensor employs four fixed anchors (at the four corners of the room, at a height of 2 m) and provides range measurements with a ranging noise standard deviation of 0.08 m. The generation frequency of sensor data is not fixed uniformly: although the program presets the UWB observation frequency to 15 Hz, in the actual simulation, the first three robots generate IMU and UWB data every 0.1 s (10 Hz), while the remaining robots generate data every 0.2 s (5 Hz); thus, the 15 Hz independent sampling is not strictly followed.

The following is the Algorithm 1:
**Algorithm 1** Pseudocode of cross-checking.  1:**while** system Running **do**  2:      (i,j,z_meas)←getMeasurement()  3:      z_pred←inverse(pose[i])∗pose[j]  4:      residual←log(inverse(z_pred)∗z_meas)  5:      graph.addEdge(i,j,z_meas,informationMatrix)  6:      **if** should Optimize() **then**  7:            optimizedPoses←optimizeGraph(graph)  8:            updateRobotPoses(optimizedPoses)  9:      **end if**10:**end while**

## 5. Intelligent Search and Search Algorithm

This section details the intelligent search and navigation algorithm based on the ant colony optimization algorithm in the project, including the dynamic grouping strategy, path decision-making, target detection, and resource reallocation.

### 5.1. Weight-Based Dynamic Grouping Strategy

#### 5.1.1. Target Priority Weight Allocation

In multi-target search tasks, different targets may have different importance levels. This paper adopts a weight allocation strategy, assigning a priority weight $w_{i}$ to each target. The weights satisfy the normalization condition:

(27) $$w_{A}+w_{B}+w_{C}=1.$$

Weight determination can be based on task requirements, target risk level, time sensitivity, etc. In the simulation of this paper, the weights for three targets are set as $w_{A}=0.35$, $w_{B}=0.325$, and $w_{C}=0.325$.

The three objectives in this paper have their own characteristic attributes. *A* hopes that the search time is the shortest, *B* hopes that the search mileage is the shortest, and *C* hopes that the search risk distance time is controllable. Because *C* may be a dangerous source, we should avoid approaching *C* but not finding *C*. Design Rationale for Weight Allocation: In this paper, the time cost of robot target search, the search mileage (energy consumption) of the robot, and the target risk level are used as the weight coefficients to determine the grouping of robots. In order to verify the weight sensitivity, five groups of typical weight combinations were selected for comparison, as shown in Table 1.

Calculation formula of the index score: The score of each index is the normalized dimensionless value, and the value range is [0,1]. The smaller the score is, the better the performance of the index is. For a set of experiments (e.g., W1–W5 and multiple runs), collect all raw data for each metric. The normalization formula is as follows:

(28) $${\mathrm{score}}_{i}=\frac{Original_{i}-\min_{Original}}{\max_{Original}-\min_{Original}}$$

where $Original_{i}$ is the original measured value of the *i*-th run or the average of a group; $\min_{Original}$ is the minimum value (optimal value) of the index in all comparison combinations; and $\max_{Original}$ is the maximum value (worst value) of the indicator in all comparison combinations.

Calculation formula of comprehensive cost: The comprehensive cost *J* is the weighted sum of the scores of the three indexes, and the weights are $w_{A}$, $w_{B}$, and $w_{C}$ of each group.

(29) $$J=\varpi_{A}\;score_{time}+\varpi_{B}\;score_{mileage}+\varpi_{C}\;score_{risk}$$

As shown in Table 2, W3 yields the lowest comprehensive cost (0.100) and the most balanced scores among the three indicators (0.100, 0.108, and 0.105), ranking first overall. This result verifies that W3 achieves the best overall performance and robustness. The comprehensive performance of W4 and W5 is close to that of W3, but still slightly inferior. In contrast, W1 has the highest comprehensive cost because of its excessive bias toward time cost, which causes significant degradation in the other two indicators.

**Figure 2:**
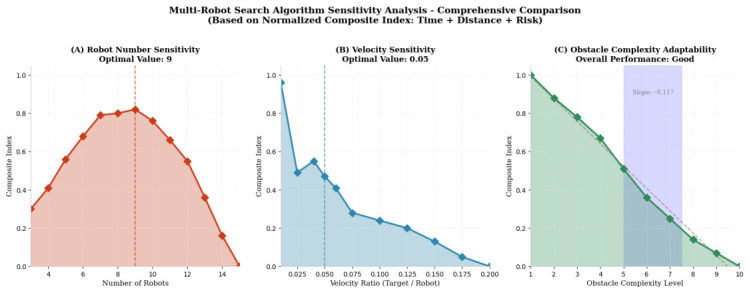
Multi-robot search algorithm sensitivity analysis—comprehensive comparison.

Based on the above weight setting for the three objectives, namely (0.35,0.325,0.325), further experiments were designed to evaluate the sensitivity to the number of robots, the sensitivity to robot speed, and the environmental adaptability of the robot formation. The results indicate the following optimal settings: 9 robots (with a peak comprehensive score of 0.812), a speed ratio of 0.05 (with an optimal comprehensive score of 0.470), and overall good environmental adaptability (with a trend slope of −0.117, which is close to zero and indicates strong adaptability). These results will be used as reference data in subsequent algorithm comparison experiments, as shown in Figure 2.

#### 5.1.2. K-Means Clustering Grouping Algorithm

Based on target priority and robot initial positions, the K-means clustering algorithm is used to divide robots into different search groups. The data is divided into *K* clusters, each represented by its center (mean vector). Optimized objective function:

(30) $$J=\sum_{i=1}^{N}\sum_{k=1}^{K}r_{ik}\;{∥x_{i}-\mu_{k}∥}^{2},$$

where $r_{ik}$ indicates whether the sample $x_{i}$ belongs to cluster *k*, and $\mu_{k}$ is the center of cluster *k*.

The algorithm steps are:

(1) Initialization: Initialize cluster centers based on target positions and weights.
(2) Assignment: Assign each robot to the nearest cluster center.
(3) Update: Recalculate the center position of each cluster.
(4) Iteration: Repeat steps 2–3 until convergence.

#### 5.1.3. Adaptive Adjustment of Group Size

In the project, an attraction matrix *A* is set, where *m* is the number of robots, *n* is the number of targets, and the element $a_{ij}$ represents the attraction of robot *i* to target *j*. The weight vector $w\in R^{n}$, satisfying $\sum_{j=1}^{n}w_{j}=1$, is used to represent the importance of each goal, and the total number of robots is *m*. Set an assignment vector assign, where assign(i)=j means that robot *i* is assigned to target *j*, and an initial value of 0 means that it is not assigned. The following is the process of the robot grouping algorithm.

1. Calculate the target capacity.

The initial capacity $c^{\left(0\right)}\in Z^{n}$ for each target is

(31) $$c_{j}^{\left(0\right)}=\max\left(1,\lfloor w_{j}m+0.5\rfloor \right),\;j=1,\ldots ,n.$$

Make the total capacity equal to the total number of robots *m* by the following iterative adjustment:

- If $\sum_{j=1}^{n}c_{j}^{\left(0\right)}>m$, repeatedly select the target $j^{*}=\arg\max_{j}c_{j}$ with the largest current capacity, and let $c_{j^{*}}\leftarrow c_{j^{*}}-1$, until the sum is equal to *m*.
- If $\sum_{j=1}^{n}c_{j}^{\left(0\right)}<m$, repeatedly select the target $j^{*}=\arg\min_{j}c_{j}$ with the current minimum capacity, and let $c_{j^{*}}\leftarrow c_{j^{*}}+1$, until the sum is equal to *m*.

Finally, the capacity vector *c* is obtained.

2. Allocate robots by goal (greedy allocation).

For each target j=1,…,n, $c_{j}$ allocations are performed in turn. The currently available robot set

(32) $$R_{\mathrm{avail}}=\left\{i|\mathrm{assign}\left(i\right)=0\right\}$$

is determined, and the robot

(33) $$i^{*}=\arg\max_{i\in R_{\mathrm{avail}}}a_{ij}$$

with the greatest attraction to target *j* is selected in $R_{\mathrm{avail}}$. If $i^{*}$ exists, let $\mathrm{assign}(i^{*})=j$, and set the $i^{*}$-th row of matrix *A* to −∞ (indicating that the robot has been assigned and will not participate in the subsequent selection).

3. Disposal of remaining robots.

For all unassigned robots i∈{i∣assign(i)=0}, the target

(34) $$j^{*}=\arg\max_{j=1,\ldots ,n}a_{ij}$$

with the largest attraction is selected, respectively, and let $\mathrm{assign}\left(i\right)=j^{*}$. Finally, the vector assign is the assignment result for each robot.

### 5.2. Path Decision-Making Based on Ant Colony Algorithm

**Figure 3:**
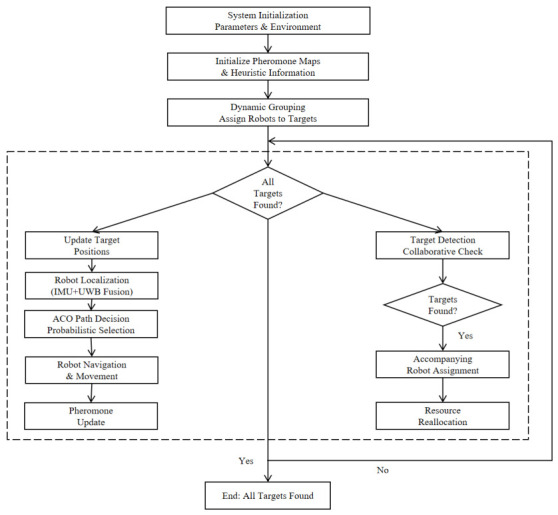
ACO algorithm flowchart.

Ant colony optimization (ACO) is a heuristic optimization algorithm that simulates the behavior of ants looking for food, as shown in Figure 3. Its core is to guide the search process through the positive feedback mechanism of pheromones. The robot releases pheromones in the process of moving, and the follow-up robot chooses the moving direction according to the pheromone concentration and heuristic information, and gradually approaches the optimal path.

#### 5.2.1. Transition Probability

At time *t*, the probability that ant *k* at node *i* selects the next node *j* is determined by

(35) $$P_{ij}^{k}\left(t\right)=\left\{\begin{array}{cc} \frac{{\left[\tau_{ij}\left(t\right)\right]}^{\alpha }{\left[\eta_{ij}\right]}^{\beta }}{\sum_{l\in {\mathrm{allowed}}_{k}}{\left[\tau_{il}\left(t\right)\right]}^{\alpha }{\left[\eta_{il}\right]}^{\beta }}, & j\in {\mathrm{allowed}}_{k},\\ 0, & \mathrm{otherwise}. \end{array}\right.$$

Among them, $\tau_{ij}\left(t\right)$ is the pheromone concentration on edge (i,j); $\eta_{ij}$ is the heuristic information and is usually taken as the reciprocal of the distance $d_{ij}$, that is,

(36) $$\eta_{ij}=\frac{1}{d_{ij}}.$$

α and β are regulating parameters which respectively control the relative importance of pheromone and heuristic information; ${\mathrm{allowed}}_{k}$ is the set of nodes that are currently allowed to be visited by ant *k* (e.g., nodes that have not been visited). The ant selects the next moving position according to this probability distribution in a roulette manner.

#### 5.2.2. Pheromone Reinforcement

Each ant releases pheromones on its constructed path, usually in the following way:

(37) $$\tau_{ij}\leftarrow \tau_{ij}+\sum_{k=1}^{m}\Delta \tau_{ij}^{k},$$

where *m* is the number of ants, and $\Delta \tau_{ij}^{k}$ is the pheromone increment left by the *k*-th ant on edge (i,j). In a common ant system,

(38) $$\Delta \tau_{ij}^{k}=\left\{\begin{array}{cc} Q/L_{k}, & \mathrm{if}\;\mathrm{ant}\;k\;\mathrm{passes}\;\mathrm{edge}\;(i,j),\\ 0, & \mathrm{otherwise}, \end{array}\right.$$

where *Q* is the pheromone intensity constant, and $L_{k}$ is the total length of the path taken by ant *k* in this iteration.

### 5.3. Target Discovery and Accompanying Mechanism

When a robot approaches a target, target confirmation is performed. The confirmation condition is as follows: the distance between the robot and the target is less than a threshold $d_{\mathrm{th}}$ and multiple robots simultaneously detect the target. This design integrates group information (detection probability) and individual perception (close range), improves the robustness of detection, and avoids misjudgment or omission of a single sensor.

After target confirmation, the following operations are executed:

(1) Select the robot closest to the target as the accompanying robot.
(2) The accompanying robot stays at the target location, continuously monitoring the target state.
(3) Release a large amount of pheromones at the target location to guide other robots.
(4) Reassign other robots from that group to search for remaining targets.

### 5.4. Resource Reallocation Strategy

**Figure 4:**
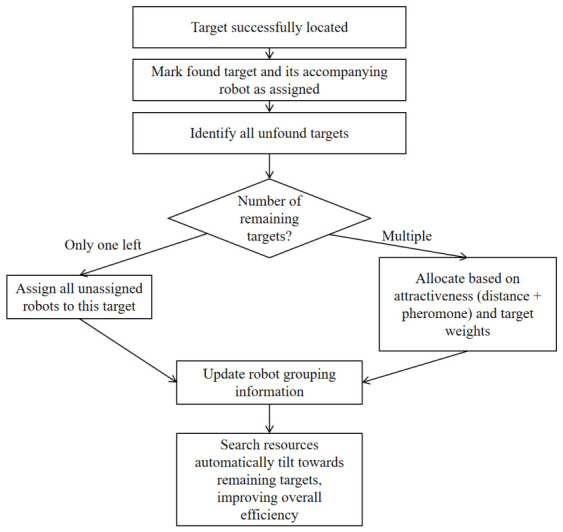
Resource reallocation strategy.

Under the algorithm framework of this project, the core of the resource reallocation strategy is to dynamically adjust the search target grouping of the robot according to the target discovery state and the current environmental pheromone distribution, so as to optimize the search efficiency of the remaining targets. The specific process is shown in Figure 4.

Trigger condition: When a target is successfully located, the system immediately calls the reallocation function to regroup_ACO. At this time, the found target and its companion robot are marked as “assigned” and no longer participate in the subsequent search. Residual target analysis: identify all targets that have not yet been found and calculate their weights (based on the initial weight normalization). If there is only one target left, all the unassigned active robots are assigned to the target to directly form a centralized search. Attraction calculation: For the multi-target case, a combined attraction is calculated for each active robot and each remaining target, which consists of two parts: distance attraction, i.e., the reciprocal of the distance between the robot’s current position and the target position, 1/(distance+1), reflecting the spatial proximity; and pheromone attraction, i.e., the normalized value of the pheromone concentration of the grid where the robot is located (the pheromone map corresponding to the current target), which represents the historical search value of the area. The comprehensive formula is

(39) $$\mathrm{attraction}=\mathrm{distance}\;\mathrm{attraction}\times \left(1+0.5\times \mathrm{pheromone}\;\mathrm{attraction}\right),$$

so that the robot not only tends to the target but also takes into account the pheromone accumulation on the path.

Intelligent assignment algorithm: The improved Hungarian greedy assignment algorithm (improved_assignment_algorithm) is used, and the steps are as follows. The robot capacity (i.e., the number to be allocated) is calculated based on each target weight. Iteratively select the robot that is currently the most attractive and unassigned to a target, and assign it to the target until its capacity is saturated. If there are any remaining robots, they are assigned to the most attractive target, ensuring that all robots are reassigned. Update grouping information: write the allocation result into the global grouping structure groups, and update the group_ID, target_ID and corresponding group name of each robot, so that its subsequent navigation decisions are based on the new target.

When the number of targets is reduced, the search resource is automatically tilted to the remaining targets, and the pheromone is used to guide the robot to explore the high-value areas preferentially, thus improving the overall search efficiency. The whole process does not rely on global planning, but is based on local information and distributed decision-making, which reflects the self-organizing characteristics of the ant colony algorithm.

## 6. Experimental Model

This section introduces the establishment of the experimental model, including environment modeling, robot motion and perception models, target characteristic analysis, and problem formalization.

### 6.1. Environment Model

The simulation environment in this paper is a 10 m × 8 m 2D planar area. Five obstacles are placed in the environment to simulate real-world obstacles like furniture and walls. The positions and sizes of the obstacles are shown in Table 3.

Four UWB anchors are placed in the environment at the four corners of the area, with coordinates: $A_{1}\left(0,0\right)$, $A_{2}\left(10,0\right)$, $A_{3}\left(10,8\right)$, $A_{4}\left(0,8\right)$. This layout provides maximum localization coverage.

### 6.2. Robot Motion Model and Sensor Noise

The robot uses a differential wheeled mobile robot model. The kinematic equations are

(40) $$\left\{\begin{array}{c} \dot{x}=v\cos\theta ,\\ \dot{y}=v\sin\theta ,\\ \dot{\theta }=\omega . \end{array}\right.$$

where (x,y) is the robot position, θ is the orientation angle, *v* is the linear velocity, and ω is the angular velocity. The robot’s maximum linear velocity is 2 m/s, and its maximum angular velocity is 1.2 rad/s.

The IMU sensor includes accelerometer and gyroscope noise. Accelerometer measurement noise standard deviation is $\sigma_{a}=0.05\;m/s^{2}$, and bias is $b_{a}={\left[0.005,0.005,0.005\right]}^{T}\;m/s^{2}$. Gyroscope measurement noise standard deviation is $\sigma_{g}=0.005\;\mathrm{rad}/s$, and bias is $b_{g}={\left[0.0005,0.0005,0.0005\right]}^{T}\;\mathrm{rad}/s$. UWB noise standard deviation is $\sigma_{d}=0.08\;m$. The UWB update frequency is 15 Hz.

### 6.3. Target Characteristic and Discovery Threshold

This project sets three targets, where target A is static, and targets B and C are dynamic. The initial positions and motion characteristics of the targets are shown in Table 4. Dynamic (0.1,0.05) and dynamic (0.05,0.1) in the table are the velocities of moving targets B and C in *X* and *Y* directions, respectively, assuming a uniform motion mode.

The target discovery threshold is set to $d_{\mathrm{th}}=0.25\;m$. When the distance between a robot and a target is less than this threshold, the target is considered discovered. For higher reliability, multiple simultaneous robot confirmations are required.

### 6.4. Problem Formalization Description

The multi-robot collaborative search problem can be formally described as follows. Given: (1) robot set $R=\{r_{1},r_{2},\ldots ,r_{N}\}$; (2) target set $T=\{t_{1},t_{2},\ldots ,t_{M}\}$; (3) search area Ω. Find: robot path planning that results in all targets being discovered in the shortest possible time. Evaluation metrics include: (1) average search time; (2) search coverage rate; (3) localization accuracy; (4) system robustness.

## 7. Simulation and Results Analysis

This section introduces the setup of the simulation platform, experimental parameter settings, and results analysis. By comparing with PSO, GWO, and random search algorithms, the effectiveness of the proposed algorithm is verified.

### 7.1. Simulation Platform and Parameter Settings

This paper uses MATLAB (R2024b, The MathWorks, Inc., Natick, MA, USA) for simulation verification. MATLAB has powerful numerical computation and visualization capabilities, making it suitable for multi-robot system simulation. The simulation environment mainly includes the environment modeling module, robot module, sensor module, ant colony algorithm module, and visualization module. The main simulation parameters are listed in Table 5 and are configured as follows.

### 7.2. The Process of Locating the Target

**Figure 5:**
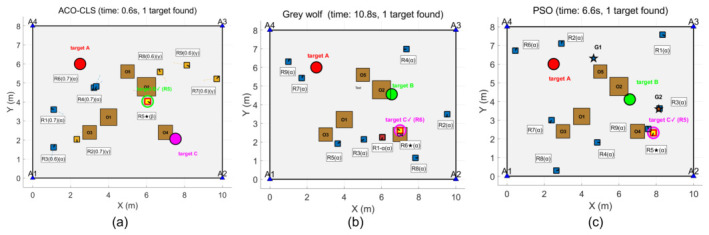
First target discovery: (**a**) ACO-CLS; (**b**) grey wolf; (**c**) PSO.

The simulation results show that the ACO-CLS search method proposed in this paper only takes 0.6 s to find the first target, which is significantly lower than the 10.8 s of the grey wolf algorithm and the 6.6 s of the PSO algorithm, and the three algorithms are the first to find the moving target, as shown in Figure 5.

**Figure 6:**
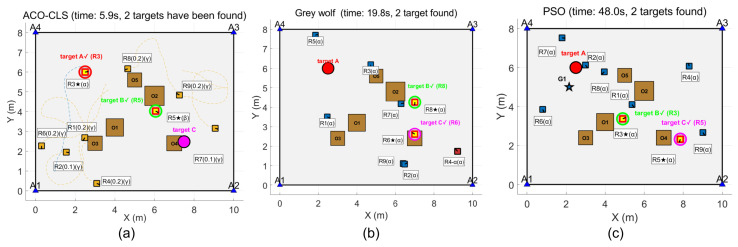
Second target discovery: (**a**) ACO-CLS; (**b**) grey wolf; (**c**) PSO.

**Figure 7:**
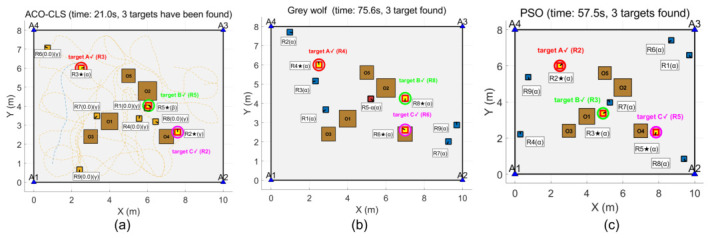
Third target discovery: (**a**) ACO-CLS; (**b**) grey wolf; (**c**) PSO.

The results are illustrated in Figure 6 and Figure 7. The ACO-CLS search method takes a total of 5.9 s to find the second target, which is significantly lower than 10.8 s of the grey wolf algorithm and 48 s of the PSO algorithm. The new method finds the fixed target this time, and the rest of the algorithm finds the moving target. The ACO-CLS search method takes a total of 21 s to find the third target, which is significantly lower than the grey wolf algorithm’s 75.6 s and the PSO algorithm’s 57.5 s.

Table 6 shows the target discovery time comparison for different algorithms. The average search time of the proposed algorithm is 21 s, significantly better than PSO (57.5 s) and GWO (75.6 s).

**Figure 8:**
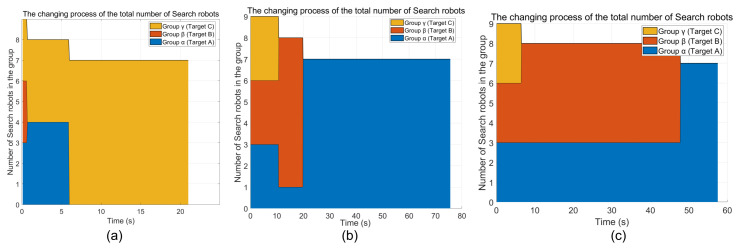
The process of changing the total number of search robots. (**a**) ACO-CLS; (**b**) grey wolf; (**c**) PSO.

The following is the change process of the total number of search robots. From Figure 8, we can see that the total number of search robots is 9 at the beginning. When a target is found, one robot is left as a companion robot, and the robots are regrouped to search for the target. The ACO-CLS is regrouped to 4 robots, the grey wolf algorithm is a group of 1 robot and a group of 7 robots, and the PSO algorithm is a group of 5 robots and a group of 3 robots. When the second target is found, the search robots are all reorganized into a group of seven robots.

**Figure 9:**
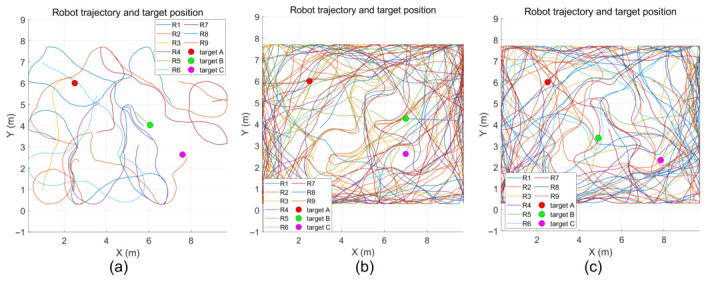
Moving track of searching target for robot. (**a**) ACO-CLS; (**b**) grey wolf; (**c**) PSO.

The following is the motion trajectory of the search robot. It can be seen from Figure 9 that the line of ACO-CLS is the simplest, indicating that the search efficiency of this method is the highest. Because of the long search time and low efficiency, the grey wolf algorithm and PSO algorithm search the whole area in a large range in turn. The search coverage rate is an important metric for evaluating the global exploration capability and efficiency of robot search. The search coverage rate of the proposed algorithm reaches 72%, lower than PSO’s 78% and GWO’s 82%. This is mainly attributed to the pheromone mechanism of the ant colony algorithm, which can improve the efficiency of robot search.

**Figure 10:**
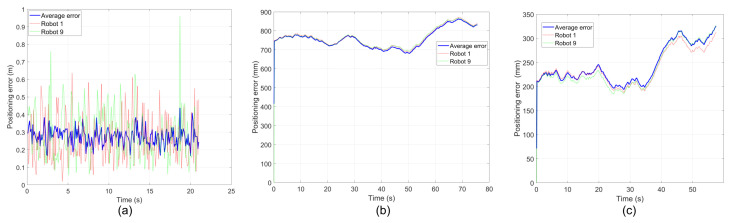
Positioning error of searching robot. (**a**) ACO-CLS; (**b**) grey wolf; (**c**) PSO.

The following is the positioning error of the search robot. It can be seen from Figure 10 that the positioning error of ACO-CLS is lower than the grey wolf method.

## 8. Conclusions

This paper addresses the problems of multi-robot collaborative localization and target search, proposing an intelligent search strategy based on the ant colony optimization algorithm. The main work and innovations include:

(1) Proposing a multi-sensor fusion localization algorithm based on IMU and UWB sensors, using ESKF to achieve high-precision position estimation. Localization accuracy reaches 0.31 m, and convergence time is about 21 s.
(2) Designing a weight-based dynamic grouping strategy and achieving intelligent grouping based on target priority and robot positions. This strategy can effectively allocate search resources and avoid waste.
(3) Introducing the ant colony algorithm into multi-robot search and guiding robot search through pheromone updates and heuristic information. Designing an intelligent reallocation mechanism after target discovery and achieving dynamic optimization of system resource allocation.

Simulation results show that the proposed algorithm outperforms traditional methods in localization accuracy, search efficiency, and system robustness, demonstrating significant theoretical value and application prospects.

Future work directions include: (1) researching algorithm performance in more complex environments; (2) introducing deep learning methods to optimize path decision-making; (3) conducting physical experiments to verify the practical performance of the algorithm.

## Figures and Tables

**Table 1:** Five groups of typical weight combinations.

Combination	$w_{A}$	$w_{B}$	$w_{C}$	Description of Characteristics
W1	0.50	0.25	0.25	Strongly biased toward target A
W2	0.40	0.30	0.30	Moderately biased toward target A
W3	0.35	0.325	0.325	Slightly biased to target A
W4	0.33	0.34	0.33	Slightly biased to target B
W5	0.33	0.33	0.34	Slightly biased toward target C

**Table 2:** Comprehensive cost of five groups of typical weights.

Combination	Weight Proportion	Time Cost Score	Mileage Score	Risk Score	Comprehensive Cost	Overall Ranking
W1	(0.50, 0.25, 0.25)	0.082	0.156	0.142	0.124	5
W2	(0.40, 0.30, 0.30)	0.091	0.131	0.123	0.108	4
W3	(0.35, 0.325, 0.325)	0.100	0.108	0.105	0.100	1
W4	(0.33, 0.34, 0.33)	0.107	0.100	0.109	0.103	2
W5	(0.33, 0.33, 0.34)	0.109	0.110	0.098	0.104	3

**Table 3:** Obstacle parameters.

Obstacle ID	*X* (m)	*Y* (m)	Size (m)
O1	4.0	3.2	0.7
O2	6.0	4.8	0.8
O3	3.0	2.5	0.9
O4	7.0	5.5	0.75
O5	5.0	6.0	0.85

**Table 4:** Target parameters.

Target	*X* (m)	*Y* (m)	Weight	Motion
A	2.5	6.0	0.35	Static
B	6.0	4.0	0.325	Dynamic (0.1,0.05)
C	7.5	2.0	0.325	Dynamic (0.05,0.1)

**Table 5:** Simulation parameters.

Parameter	Value	Description
Room Size	10 m × 8 m	Search area
Number of Robots	9	Mobile robots
Number of Targets	3	Search targets
Number of Obstacles	5	Static obstacles
Number of Anchors	4	UWB base stations
Simulation Time	400 s	Max duration
Time Step	0.1 s	Discretization
ACO Alpha	1.5	Pheromone weight
ACO Beta	2.5	Heuristic weight
ACO Rho	0.1	Evaporation rate

**Table 6:** Algorithm comparison results.

Algorithm	Search Time (s)	Error (m)
ACO (Proposed)	21	0.31
GWO	75.6	0.78
PSO	57.5	0.26

## Data Availability

The data presented in this study are available on request from the corresponding author.
